# Developmental Fluoride Neurotoxicity: Choi et al. Respond

**DOI:** 10.1289/ehp.1206192R

**Published:** 2013-03-01

**Authors:** Anna L. Choi, Philippe Grandjean, Guifan Sun, Ying Zhang

**Affiliations:** 1 Harvard School of Public Health, Boston, Massachusetts, E-mail: achoi@hsph.harvard.edu; 2 China Medical University, Shenyang, China

Sabour and Ghorbani’s comments about the reported mean difference in IQ (intelligence quotient) scores reported in our article (Choi et al. 2012) suggest a misunderstanding of the scale unit we used and the public health significance of even a small decrease in the average IQ associated with exposure. We appreciate this opportunity to clarify the factual information about the reported IQ measure.

The standardized weighted mean difference (SMD) in IQ score between exposed and reference populations was –0.45 (95% confidence interval: –0.56, –0.35) using a random-effects model (Choi et al. 2012). We used the SMD because the studies we included used different scales to measure the general intelligence. The SMD is a weighted mean difference standardized across studies, giving the average difference in standard deviations for the measure of that outcome. For commonly used IQ scores with a mean of 100 and an SD of 15, 0.45 SDs is equivalent to 6.75 points (rounded to 7 points). As research on other neurotoxicants has shown, a shift to the left of IQ distributions in a population will have substantial impacts, especially among those in the high and low ranges of the IQ distribution (Bellinger 2007).
